# Composite lipid indices in patients with obstructive sleep apnea: a systematic review and meta-analysis

**DOI:** 10.1186/s12944-023-01859-3

**Published:** 2023-06-29

**Authors:** Amir Hossein Behnoush, Pegah Bahiraie, Zahra Shokri Varniab, Laleh Foroutani, Amirmohammad Khalaji

**Affiliations:** 1 School of Medicine, Tehran University of Medical Sciences, Tehran, Iran; 2 Non-Communicable Diseases Research Center, Endocrinology and Metabolism Population Sciences Institute, Tehran University of Medical Sciences, Tehran, Iran; 3 School of Medicine, Shahid Beheshti University of Medical Sciences, Tehran, Iran; 4 Pediatric Urology and Regenerative Medicine Research Center, Gene, Cell and Tissue Research Institute, Children’s Medical Center, Tehran University of Medical Sciences, Tehran, Iran

**Keywords:** Lipids, Obstructive sleep apnea, Atherogenic index of plasma, Lipid accumulation product, Visceral adiposity index, Systematic review, Meta-analysis

## Abstract

**Background:**

One of the most prevalent sleep disorders affecting the individual’s daily life is obstructive sleep apnea (OSA), for which obesity is a major risk factor. Several novel lipid indices have been suggested to have associations with OSA, among which visceral adiposity index (VAI), atherogenic index of plasma (AIP), and lipid accumulation product (LAP) are the most important ones. Herein, the current study aimed to systematically investigate the association between these indices and OSA.

**Methods:**

Four international databases, including PubMed, Scopus, the Web of Science, and Embase were searched in order to find relevant studies that investigated LAP, VAI, or AIP in OSA and compared them with non-OSA cases or within different severities of OSA. Random-effect meta-analysis was used to generate the standardized mean difference (SMD) and 95% confidence interval (CI) of the difference in lipid indices between OSA and non-OSA cases. Moreover, the pooled area under the receiver operating characteristic curves (AUCs) observed in individual studies for diagnosis of OSA based on these lipid indices were calculated by random-effect meta-analysis.

**Results:**

Totally 14 original studies were included, comprised of 14,943 cases. AIP, LAP, and VAI were assessed in eight, five, and five studies, respectively. Overall, these lipid indices had acceptable diagnostic ability (AUC 0.70, 95% CI 0.67 to 073). Meta-analysis revealed that AIP was significantly higher in patients with OSA (SMD 0.71, 95% CI 0.45 to 0.97, *P* < 0.01). Moreover, AIP also increased in higher severities of OSA. Regarding LAP, a higher LAP was observed in OSA/patients with high risk for OSA rather than in controls/low risk for OSA (SMD 0.53, 95% CI 0.25 to 0.81, *P* < 0.01). VAI was also increased in OSA based on results from two studies.

**Conclusion:**

These findings suggest that composite lipid indices are increased in OSA. Also, these indices can have the potential beneficiary diagnostic and prognostic ability in OSA. Future studies can confirm these findings and enlighten the role of lipid indices in OSA.

**Supplementary Information:**

The online version contains supplementary material available at 10.1186/s12944-023-01859-3.

## Introduction

Obstructive sleep apnea (OSA) is defined by alternating episodes of hypopnea and breathing stops during sleep [1]. According to a health survey of the general population, 10.0% of women and 20.2% of men in the general population suffer from moderate-to-severe OSA [2]. OSA tends to develop more frequently in overweight or obese individuals, and obesity is a leading OSA risk factor, as is increased visceral fat [3, 4]. As a consequence of obesity, there are several mechanisms that can lead to OSA, such as inflammation, the collapsibility of the pharyngeal airway due to excessive fat deposition, oxidative stress, and even mutual interactions between the adipose tissue and the respiratory system [5].

OSA is commonly recognized as cardiovascular morbidity and mortality risk factor [6]. A number of factors contribute to this association, including sympathetic activity, systemic inflammation and oxidative stress, hypertension, obesity, and dyslipidemia resulting from OSA [7–9]. Sleep fragmentation or sleep restriction leads to an increase in food intake and the tendency to consume fat-rich foods, which contributes to weight gain as a result [5, 10]. Several mechanisms can contribute to dyslipidemia caused by OSA [10, 11].

Patients with heart disease have a higher prevalence of OSA. Also, cardiovascular diseases (CVDs) are better predicted by visceral fat distribution than by body mass index (BMI) [12, 13]. In addition to classic blood lipids commonly measured in patients, several novel composite lipid indices have been suggested to have implications in CVDs and even better predictors of these diseases [14, 15]. This link between these lipid indices and CVDs can also be observed in OSA. They include three main lipid indices including lipid accumulation product (LAP) calculated from triglyceride (TG) and waist circumference (WC), atherogenic index of plasma (AIP) composed of high-density lipoprotein cholesterol (HDL-C) and TG, and visceral adiposity index (VAI) calculated from WC, body mass index (BMI), TG, and HDL-C.

With consideration, CVD is highly common among OSA patients, and dyslipidemia and obesity are important factors for CVD; however, to the best of our knowledge, the correlation between composite lipid indices and OSA has not been systematized. Moreover, although several diagnostic and prognostic biomarkers have been investigated in OSA [16–19], there is still a lack of suitable clinically-compatible biomarkers for OSA. Several studies have evaluated the association between VAI, LAP, and AIP with OSA [5, 7, 20]. The goal of this study was to assess whether the composite lipid indices are independently associated with OSA and whether there are any differences in composite lipid indices between OSA patients and controls. The findings of this study could be potentially useful in clinical settings in order to help diagnose and determine the prognosis of OSA.

## Methods

### Protocol

This study adhered to Preferred Reporting Items for Systematic Reviews and Meta-Analyses (PRISMA) 2020 protocol [21]. The study’s protocol was registered in the PROSPERO registry (registration code: CRD42023422039).

### Search strategy and databases

The four major international databases, Scopus, Medline (PubMed), Embase, and Web of Science (ISI), were comprehensively searched on 11 May 2023. The search syntax was composed within each database using keywords and common acronyms for "obstructive sleep apnea" such as "sleep-disordered breathing" OR "OSA" OR "SAS" OR "SDB" combined with word alterations of "lipid indices" such as "atherogenic index" OR "visceral adiposity index" OR "lipid accumulation product" OR "atherogenic index of plasma" (supplementary table 1). The related MeSH terms (for PubMed) and Emtree terms (for Embase) were also used to broaden the search results. A manual search was also conducted within the references of the included articles to include any missing relevant studies.

### Eligibility criteria

Inclusion criteria consisted of articles that met all of the following items: (1) prospective, retrospective, or cross-sectional studies on human subjects with OSA; (2) written in English; (3) assessed at least one of the target composite lipid indices including VAI, LAP, or AIP and compared them between OSA and non-OSA cases or within different severities of OSA, characterized by apnea–hypopnea index (AHI) [22]. Moreover, studies assessing the relationship between complications and prognosis of patients with OSA with lipid indices were included. These lipid indices are calculated as follows:

$${\varvec{A}}{\varvec{I}}{\varvec{P}}={log}_{10}\left(\frac{TG}{HDL}\right)$$

$${\varvec{V}}{\varvec{A}}{\varvec{I}}\boldsymbol{ }({\varvec{M}}{\varvec{a}}{\varvec{l}}{\varvec{e}})=\boldsymbol{ }\frac{WC(cm)}{39.68+(1.88\times BMI\left({}^{kg}\!\left/ \!{}_{{m}^{2}}\right.\right))}\times \frac{TG({}^{mmol}\!\left/ \!{}_{L}\right.)}{1.03}\times \frac{1.31}{HDL({}^{mmol}\!\left/ \!{}_{L}\right.)}$$

$${\varvec{V}}{\varvec{A}}{\varvec{I}}\boldsymbol{ }({\varvec{F}}{\varvec{e}}{\varvec{m}}{\varvec{a}}{\varvec{l}}{\varvec{e}})=\boldsymbol{ }\frac{WC(cm)}{36.58+(1.89\times BMI\left({}^{kg}\!\left/ \!{}_{{m}^{2}}\right.\right))}\times \frac{TG({}^{mmol}\!\left/ \!{}_{L}\right.)}{0.81}\times \frac{1.52}{HDL({}^{mmol}\!\left/ \!{}_{L}\right.)}$$

$${\varvec{L}}{\varvec{A}}{\varvec{P}}\boldsymbol{ }\left({\varvec{M}}{\varvec{a}}{\varvec{l}}{\varvec{e}}\right)=(WC\left(cm\right)-65)\times TG({}^{mmol}\!\left/ \!{}_{L}\right.)$$

$${\varvec{L}}{\varvec{A}}{\varvec{P}}\boldsymbol{ }\left({\varvec{F}}{\varvec{e}}{\varvec{m}}{\varvec{a}}{\varvec{l}}{\varvec{e}}\right)=(WC\left(cm\right)-58)\times TG({}^{mmol}\!\left/ \!{}_{L}\right.)$$

The exclusion criteria were 1) non-OSA articles; 2) reviews; 3) studies not reporting any composite lipid indices; 4) conference abstracts; 5) animal or in vitro studies.

### Selection of studies and data extraction

Two reviewers (AHB and AK) independently scrutinized titles and abstracts and retrieved records based on the eligibility criteria. Consecutively, the selected articles went through full-text evaluation for inclusion in the review. In case of any disagreement, a third researcher (PB) intervened and made the final decision. The data extraction sheet included the first author’s name, any of the present composite lipid indices values (AIP, LAP, and VAI), the diagnostic ability of composite lipid indices (area under the receiver operating characteristic curves (AUCs), sensitivity, specificity, and cut-off), the population characteristics in each arm (OSA patients and controls), the sample size in each arm, the year of publication, the design of the study, mean age, BMI, and male percentage, and key qualitative and quantitative findings.

### Quality assessment

The scoring system of the Newcastle Ottawa scale (NOS) [23] was implemented to appraise the quality of the non-randomized included studies in three domains of selection, comparability, and outcome ascertainment. In case any of the included studies had low quality (total NOS score of < 3), they were excluded from quantitative synthesis.

### Statistical analysis

STATA version 17 (Stata Corp, College Station, TX) was used to conduct meta-analyses under a *P* of 0.05 as the statistical significance level. Given the varied measurement method for each of the lipid indices, the standardized mean difference (SMD) and 95% confidence intervals (CI) were calculated using random-effect meta-analysis (restricted maximum likelihood, REML) to make uniform comparisons [24]. In order to pool the diagnostic abilities of lipid indices, we performed random-effect meta-analyses (DerSimonian and Laird) to provide forest plots for AUCs (overall AUC and 95% CI) [25]. The heterogeneity across the included records was assessed using the Cochrane Q test and *I*^*2*^ index. *I*^*2*^ values greater than 50% considered moderate-to-high heterogeneity, and *P* < 0.01 was considered statistically significant for Q-statistics. Publication bias was assessed with Begg's test, Egger's test, and funnel plot asymmetry [26]. Finally, sensitivity analysis by leave-one-out was performed to investigate the effect of each study on the overall effect size.

## Results

### Literature search and baseline characteristics

**Fig. 1:**
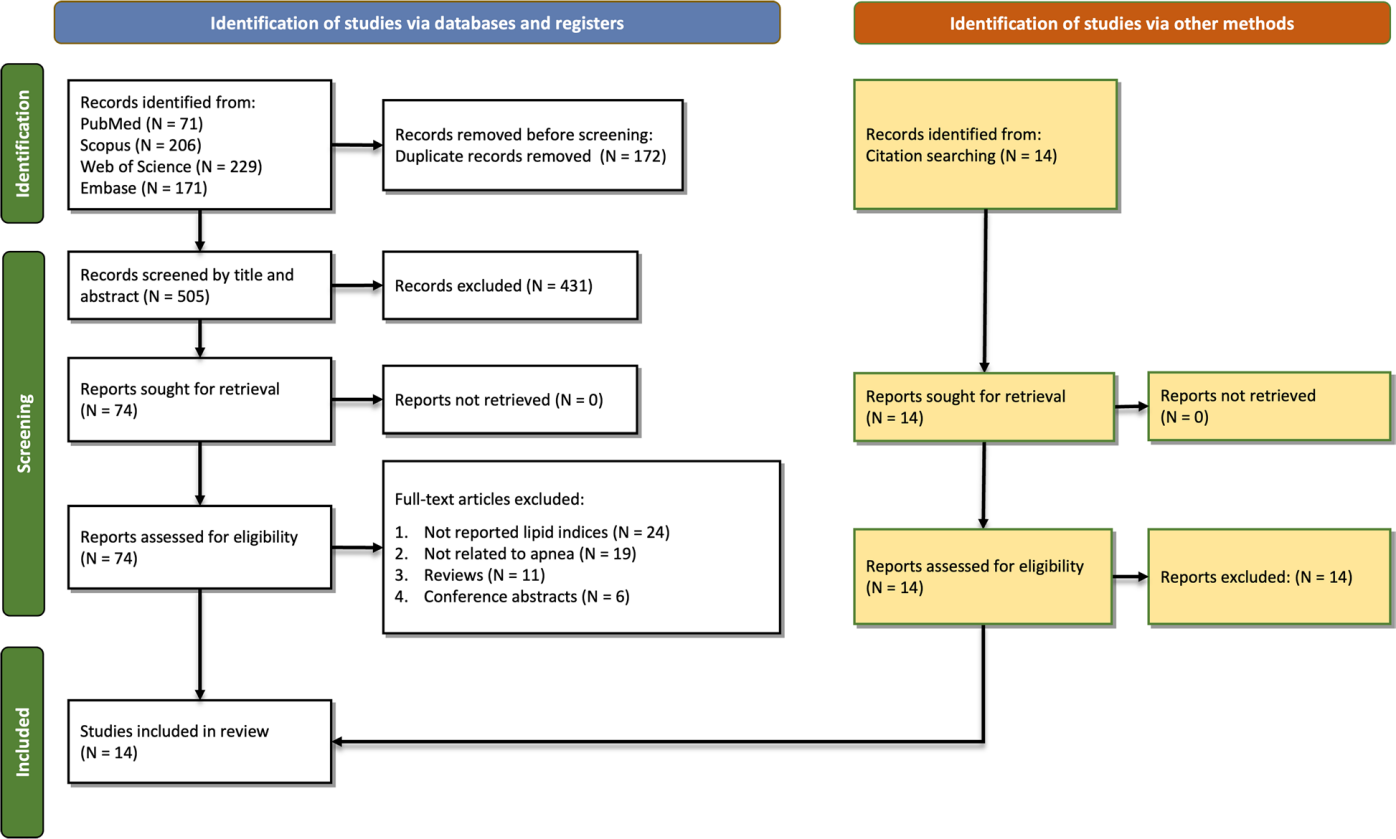
PRISMA diagram showing the search and screening process

A total of 677 records were identified from databases (Scopus: 206, PubMed: 71, Web of Science: 229, and Embase: 171). After removing 172 duplicates, 505 records were screened by abstracts and titles of which 431 were excluded. Sixty records were removed after full-text screening due to the following reasons: not reporting lipid indices (*N* = 24), not related to OSA (*N* = 19), reviews (*N* = 11), or conference abstracts (*N* = 6). Finally, 14 original studies were included in this study [5, 7, 8, 20, 27–36]. Figure 1 illustrates the selection process. After excluding patients in the study by Bikov et al. [28], because patients were similar to another study published in 2022 [7], 14,943 cases (OSA and non-OSA) were evaluated in included studies. Eight studies evaluated AIP [7, 8, 28, 29, 31–33, 35], while five studies evaluated LAP [5, 7, 27, 34, 36], and five studies evaluated VAI [7, 20, 30, 34, 36]. Table 1 summarizes the details of the studies along with their main findings. All studies had high quality with regard to the NOS system, details of which are shown in supplementary table 2.

**Table 1:** Baseline characteristics of included studies assessing composite lipid indices in OSA

Author	Year	Design	Lipid index	Populations		Sample Size		Age		BMI Total	% Male total	Findings
				**Main population**	**Control population**	**N Total**	**N Control**	**Age Total**	**Age control**			
Bianchi et al	2014	Cross-sectional	LAP	Elderly patients with aortic aneurysms and high risk for OSA	Elderly patients with aortic aneurysms and low risk for OSA	302	119	73.4 (8.6)	74.7 (8.8)	29.6 (6.2)	86.5	LAP was significantly higher in patients with higher risk of OSA (50.5 (35.5) vs. 36.7 (28.3), *P* < 0.001)
Bikov et al	2021	Cross-sectional	AIP	Patients with a diagnosis of OSA (AHI ≥ 5)	Non-OSA individuals	560	99	52.7 (13.4)	46.7 (18.8)	30.7 (5.5)	58.9	AIP was significantly higher in patients with OSA compared to controls (0.18 [0.03 to 0.31] vs. -0.15 [-0.34 to 0.06], *P* < 0.001)
Bikov et al	2022	Cross-sectional	AIP, LAP, VAI	OSA patients with mild (AHI: 5–14.9), moderate (AHI 15–29.9), and severe (AHI ≥ 30)	Non-OSA control volunteers	806	139	53.6 (13.0)	45.2 (14.6)	32.8 (6.0)	66.4	AIP, LAP, and VAI were all significantly different between the OSA group and healthy controls (*P* < 0.01)
Cai et al	2022	Cross-sectional	AIP	Hypertensive patients with OSA	NA	2281	NA	49.5 (10.4)	NA	28.4 (3.8)	68.5	There was a J-shaped relationship between AIP and new-onset MI
Cao et al	2020	Retrospective cohort	AIP	OSA patients with mild (AHI: 5–14.9), moderate (AHI 15–29.9), and severe (AHI ≥ 30)	Non-OSA controls	284	28	45.3 (11.1)	48.1 (12.6)	27.2 (3.1)	89.8	AIP was significantly different between all groups (*P* < 0.001)
Chen et al	2016	Cross-sectional	VAI	Patients with diagnosis of OSA based on AHI (mild, moderate, and severe)	Non-OSA patients (AHI 0–5)	411	50	48.8 (13.6)	44.4 (16.2)	26.5 (3.0)	75.9	There was a significant difference in terms of VAI between controls and severe OSA, mild OSA and severe OSA, and also moderate OSA and severe OSA (*P* < 0.05)
Dong et al	2020	Cross-sectional	LAP	Type 2 diabetic patients with OSA	Type 2 diabetic patients without OSA	317	112	52.4 (14.1)	47.1 (14.8)	27.9 (4.1)	69.1	With a *P* of < 0.01, a significant difference was observed between OSA and non-OSA individuals
Kim et al	2020	Retrospective cohort	AIP	Patients with a diagnosis of OSA based on AHI (mild, moderate, and severe)	Non-OSA individuals	180	NR	48.6 (13.8)	NR	26.4 (4.1)	73.9	AIP was significantly associated with OSA (*P* = 0.022); however, it was not an independent risk factor for OSA
Mazzuca et al	2014	Retrospective cohort	VAI	Patients with diagnosis of OSA	Non-OSA controls	528	55	51.3 (12.8)	45.9 (15.4)	31.0 (6.2)	80.1	VAI was not different between severities of OSA (*P* = 0.154)
Meszaros et al	2021	Cross-sectional	AIP	Patients with diagnosis of OSA	Non-OSA controls	76	30	48.9 (14.0)	41.2 (16.3)	28.2 (8.0)	50.0	Patients with OSA had significantly higher AIP compared to non-OSA ones
Otelea et al	2021	Cross-sectional	AIP	Patients with asthma and OSA	Patients with asthma without OSA	97	26	53.4 (6.8)	51.0 (4.9)	34.3 (7.1)	69.1	AIP values were significantly higher in OSA patients compared to patients with asthma (*P* = 0.04)
Wei et al	2021	Cross-sectional	LAP, VAI	Patients with diagnosis of OSA	Non-OSA controls	4747	909	NR	NR	NR	NR	LAP and VAI were higher in OSA patients compared to non-OSA individuals
Wysocki et al	2016	Prospective cohort	AIP	OSA patients (mild, moderate, and severe) without previous treatment	NA	211	NA	50.4 (12.7)	NA	30.4 (5.4)	83.4	AIP values were significantly higher in higher severities of OSA (*P* = 0.0121)
Zou et al	2020	Cross-sectional	LAP, VAI	Patients with diagnosis of OSA	Non-OSA controls	4703	890	42.3 (13.1)	NR	26.4 (3.5)	80.0	LAP showed moderate efficiency as a screening tool for OSA

Data are presented as mean (standard deviation), median [interquartile range], or percentage

*Abbreviations*: *AIP* Atherogenic index of plasma, *LAP* Lipid accumulation product, *VAI* Visceral adiposity index, *BMI* Body mass index, *MI* Myocardial infarction, *OSA* Obstructive sleep apnea, *AHI* Apnea–hypopnea index, *NR* Not reported, *NA* Not available

### Diagnostic accuracy of lipid indices in OSA

**Fig. 2:**
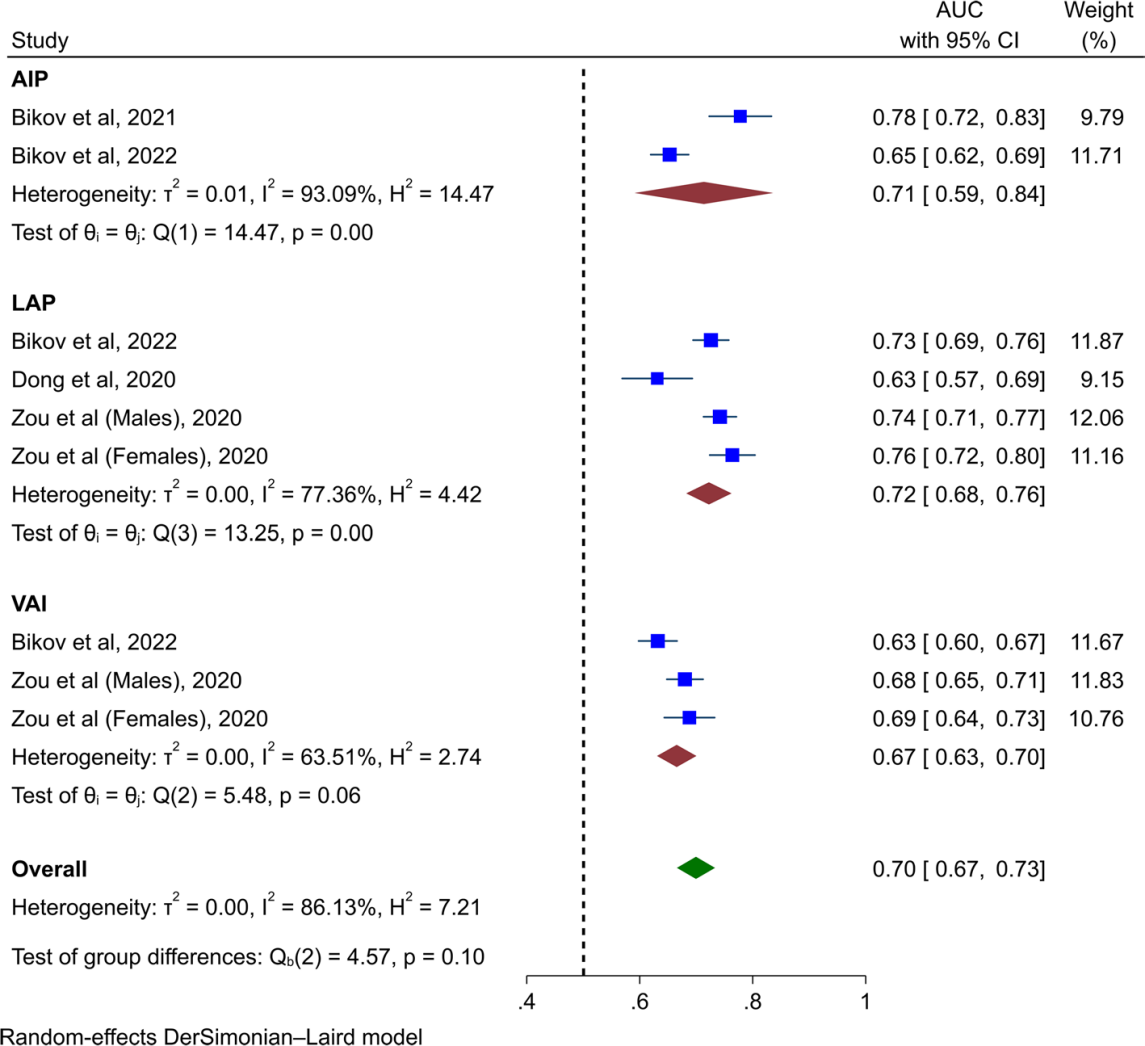
Forest plot for pooling the AUCs for diagnosis of OSA

Four studies evaluated the diagnostic ability of AIP, LAP, and VAI in distinguishing OSA patients from the controls (Table 2) [5, 7, 28, 36]. In the meta-analysis of AUCs, acceptable discrimination for composite lipid indices was found (AUC 0.70, 95% CI 0.67 to 0.73, Fig. 2). Subgroup analyses found acceptable diagnostic ability based on AUC for AIP (AUC 0.71, 95% CI 0.59 to 0.84), LAP (AUC 0.72, 95% CI 0.68 to 0.76), and VAI (AUC 0.67, 95% CI 0.63 to 0.70). Sensitivity analysis found that these results are accurate (Supplementary Fig. 1).

**Table 2:** Diagnostic ability of composite lipid indices

Author	Year	Lipid index	N total	AUC [95% CI]	Sensitivity (%)	Specificity (%)	Cut-off
Bikov et al	2021	AIP	560	0.778 [0.723 – 0.833]	NR	NR	NR
Bikov et al	2022	AIP	806	0.653 [0.619 – 0.686]	NR	NR	NR
		LAP	806	0.726 [0.694 – 0.757]	63	76	76.4
		VAI	806	0.632 [0.598 – 0.666]	NR	NR	NR
Dong et al	2020	LAP	317	0.631 [0.569 – 0.693]	NR	NR	40.77
Zou et al	2020	LAP (males)	2413	0.742 [0.713 – 0.771]	77.8	57.8	33.15
		LAP (females)	570	0.764 [0.724 – 0.804]	75.9	68.1	28.78
		VAI (males)	2413	0.680 [0.648 – 0.712]	62.2	66.2	1.91
		VAI (females)	570	0.688 [0.643 – 0.732]	68.9	61.9	1.73

Data are presented as the area under the receiver operating characteristic curve [95% confidence interval] or percentage

*Abbreviations*: *AIP* Atherogenic index of plasma, *LAP* Lipid accumulation product, *VAI* Visceral adiposity index, *AUC* Area under the receiver operating characteristic curve, *CI* Confidence interval, *NR* Not reported

### Atherogenic index of plasma (AIP) in OSA

Eight studies with 3,935 individuals evaluated AIP as a composite lipid index in OSA [7, 8, 28, 29, 31–33, 35] of which five were cross-sectional studies [7, 8, 28, 32, 33], two were retrospective cohorts [29, 31] and one was a prospective cohort [35].

#### Meta-analysis of AIP between patients with OSA and controls

**Fig. 3:**
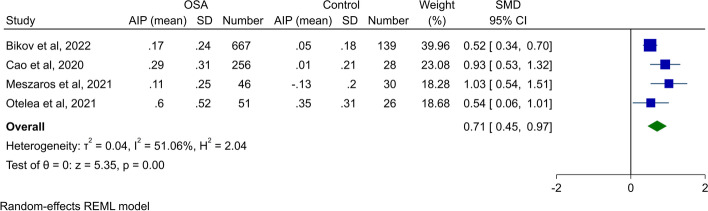
Forest plot showing meta-analysis of AIP in patients with OSA vs. healthy controls

Five studies reported AIP values in OSA patients and healthy controls [7, 28, 29, 32, 33]; however, as the population in two studies conducted by Bikov et al. [7, 28] were similar, we included one with a larger sample size [7]. Meta-analysis of four studies assessing AIP between OSA patients and controls revealed that OSA patients had significantly higher AIP (SMD 0.71, 95% CI 0.45 to 0.97, *P* < 0.01, Fig. 3). This was associated with moderate heterogeneity. Assessment of publication bias in this study showed apparent asymmetry in the funnel plot (Supplementary Fig. 2); however, Begg’s and Egger’s tests were not significant (*P* = 0.31 and 0.19, respectively). Sensitivity analysis did not show any significant difference by removal of each study (Supplementary Fig. 3).

#### AIP in different severities of OSA

Bikov et al. [28] found progressively higher AIP in higher severities (from mild to severe OSA) of OSA (*P* < 0.001). In another study by Bikov et al. [7] moderate and severe OSA were associated with significantly higher AIP compared to controls; however, AIP was comparable between mild OSA and controls (controls: 0.02 [interquartile range (IQR) -0.05 to 0.19], mild OSA: 0.13 [IQR -0.07 to 0.27], moderate OSA: 0.15 [IQR -0.04 to 0.28], and severe OSA: 0.22 [IQR 0.05 to 0.35]). In a retrospective cohort, Cao et al. [29] found significantly higher AIP values in higher severities of OSA (controls: 0.01 ± 0.21, mild OSA: 0.14 ± 0.30, moderate OSA: 0.24 ± 0.33, and severe OSA: 0.36 ± 0.30; *P* < 0.001). Kim et al. [31] used AIP tertiles and compared the prevalence of OSA and disease severity in tertiles of AIP. They found a significantly higher prevalence of OSA higher tertiles of AIP (tertile I (lowest AIP): 73.8%, tertile II: 86.4%, and tertile IIII (highest AIP): 91.7%; *P* = 0.022). Moreover, the prevalence of severe OSA and moderate OSA increased in tertile III compared to tertiles I and II (severe OSA: 32.8%, 44.1%, and 45.0%; moderate OSA: 16.4%, 20.3%, and 23.3%). Finally, Wysocki et al. [35] found a similar trend for AIP values in OSA severities. They found significantly higher AIP values in higher severities of OSA (mild: 0.35 ± 0.21, moderate: 0.36 ± 0.26, and severe: 0.48 ± 0.30).

#### Utility of AIP in detecting comorbidities in OSA patients

Bikov et al. [28] found the following AUCs for AIP in predicting 1) cerebrovascular and cardiovascular diseases (0.604 [95% CI 0.558 to 0.649]), 2) diabetes (0.627 [95% CI 0.581 to 0.671]), and 3) arterial hypertension (0.553 [95% CI 0.506 to 0.599]) in OSA patients. Another study by Bikov et al. [7] found AUCs of 0.582 [95% CI 0.543 to 0.620] for detecting hypertension, 0.533 [95% CI 0.494 to 0.572] for ischemic heart disease, and 0.602 [95% CI 0.564 to 0.640] for diabetes in OSA patients. Cai et al. [8] evaluated the correlation between this index and new-onset myocardial infarction (MI) in 2,281 OSA patients with hypertension. They found a J-shaped correlation between this index and new-onset MI with a hazard ratio of 1.42 [95% CI 1.22 to 1.65, *P* < 0.01] for a 1-standard deviation increase in AIP, which remained significant after adjusting for covariates.

### Lipid accumulation product (LAP) in OSA

Five studies with 10,875 individuals evaluated the role of LAP in OSA which all were cross-sectional studies [5, 7, 27, 34, 36].

#### Meta-analysis of LAP between patients with OSA and controls

**Fig. 4:**
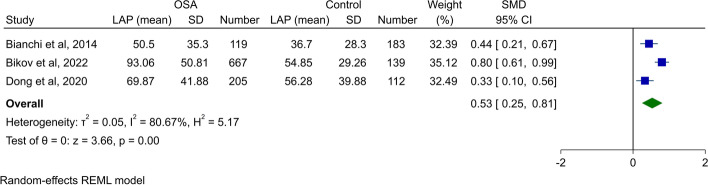
Forest plot showing meta-analysis of LAP in patients with OSA/high-risk for OSA vs. healthy controls/low-risk for OSA

Two studies evaluated LAP values between OSA patients and healthy controls [5, 7], while Bianchi et al. compared LAP between patients with low-risk or high-risk for OSA [27]. A meta-analysis of three studies between OSA/high-risk for OSA and non-OSA/low-risk for OSA found significantly higher LAP levels in patients with OSA/high-risk for OSA (SMD 0.53, 95% CI 0.25 to 0.81, *P* < 0.01, Fig. 4). The heterogeneity was high (*I*^*2*^: 80.7%). Although the funnel plot did not show any asymmetry for publication bias (Supplementary Fig. 4) and Begg’s test was not significant, Egger’s test was significant for publication bias (*P* < 0.01). Sensitivity analysis found that the primary results are accurate (Supplementary Fig. 5).

#### LAP in different severities of OSA

Bikov et al. [7] found that mild, moderate, and severe OSA was associated with significantly higher AIP compared to control (controls: 53.35 [IQR 35.95 to 75.01], mild OSA: 75.15 [IQR 40.24 to 116.24], moderate OSA: 79.02 [IQR 49.68 to 109.60], and severe OSA: 101.16 [IQR 71.98 to 143.26]; *P* < 0.05 for comparing all severities with controls).

#### Utility of LAP in detecting individuals at risk for OSA

Bianchi et al. [27] used the Berlin questionnaire to assess the risk of OSA in patients with abdominal aortic aneurysms. LAP was significantly higher in individuals who were at high risk for developing OSA compared to low-risk individuals (high-risk: 50.5 ± 35.3 compared to low-risk: 36.7 ± 28.3; *P* < 0.001).

#### Utility of LAP in diagnosing OSA

In a cross-sectional study, Bikov et al. [7] found an AUC of 0.726 [95% CI 0.694 to 0.757] for detecting OSA. Dong et al. [5] used the cut-off of 40.77 and found an AUC of 0.631 [95% CI 0.569 to 0.693] in detecting moderate-to-severe OSA. In another study, Zou et al. [36] found an AUC of 0.742 [95% CI 0.713 to 0.771] with a cut-off of 33.15 in males for predicting OSA while the AUC was 0.764 [95% CI 0.724 to 0.804] with a cut-off of 28.78 in females in predicting OSA.

#### Utility of LAP in detecting comorbidities in patients with OSA

The study by Bikov et al. [7] found AUCs of 0.658 [95% CI 0.620 to 0.694] for detecting hypertension, 0.576 [95% CI 0.537 to 0.614] for ischemic heart disease, and 0.666 [95% CI 0.628 to 0.702] for diabetes in OSA patients. Wei et al. [34] used LAP as a marker of insulin resistance in OSA patients. They found an AUC of 0.728 (cut-off: 30.16) in normal-weight OSA patients and an AUC of 0.698 (cut-off: 57.67) in overweight/obese OSA patients for predicting insulin resistance in OSA patients.

### Visceral adiposity index (VAI) in OSA

Five studies with 11,195 individuals assessed the association between VAI and OSA [7, 20, 30, 34, 36] of which four were cross-sectional studies [7, 30, 34, 36] and one was a retrospective cohort [20].

### VAI between OSA patients and controls

Two studies by Bikov et al. [7] and Chen et al. [30] reported VAI levels between OSA and controls. Bikov et al. [7] found significantly higher VAI in OSA compared to controls (2.50 [IQR 1.65 to 3.48] vs. 1.82 [IQR 1.32 to 2.50], *P* < 0.01). In line with this study, Chen et al. [30] found higher VAI levels in mild (1.90 [IQR 1.25 to 2.89]), moderate (1.93 [IQR 1.25 to 3.01]), and severe (2.53 [IQR 1.70 to 3.79]) OSA compared to controls (1.46 [IQR 0.99 to 2.75]). This difference was significant between controls and severe OSA.

#### VAI in different severities of OSA

Bikov et al. [7] found that moderate and severe OSA was associated with significantly higher VAI compared to controls; however, VAI was comparable between mild OSA and controls (controls: 1.82 [IQR, 1.32 to 2.50], mild OSA: 2.38 [IQR, 1.35 to 3.32], moderate OSA: 2.21 [IQR 1.46 to 3.22], and severe OSA: 2.63 [IQR 1.80 to 3.54]). Chen et al. [30] found higher VAI in higher severities of OSA (controls: 1.46 [IQR 0.99 to 2.75], mild OSA: 1.90 [IQR 1.25 to 2.89], moderate OSA: 1.93 [IQR 1.25 to 3.01], and severe OSA: 2.53 [IQR 1.70 to 3.79]; *P* < 0.05 for controls vs. severe, moderate vs. severe, and mild vs. severe). Mazzuca et al. [20] found insignificantly higher VAI values in severe OSA compared to mild-to-moderate OSA (control males: 1.78 ± 1.10, control females: 1.41 ± 0.61, mild-to-moderate OSA males: 1.95 ± 1.33, mild-to-moderate OSA females: 1.84 ± 1.26, severe OSA males: 2.26 ± 1.72, severe OSA females: 2.11 ± 1.18; *P* = 0.154). Moreover, they found no difference in VAI between males and females (*P* = 0.698).

#### Utility of VAI in Diagnosing OSA

In a cross-sectional study, Bikov et al. [7] found an AUC of 0.632 [95% CI 0.598 to 0.666] for detecting OSA. Zou et al. [36] found an AUC of 0.680 [95% CI 0.648 to 0.712] with a cut-off of 1.91 in males for predicting OSA while the AUC was 0.688 [95% CI 0.643 to 0.732] with a cut-off of 1.73 in females in predicting OSA.

#### Utility of VAI in detecting comorbidities in patients with OSA

The study by Bikov et al. [7] found AUCs of 0.582 [95% CI 0.543 to 0.620] for detecting hypertension, 0.538 [95% CI 0.499 to 0.577] for ischemic heart disease, and 0.608 [95% CI 0.569 to 0.645] for diabetes in OSA patients. Chen et al. [30] tried to predict the existence of metabolic syndrome in patients with OSA using VAI. They found AUC of 0.836 [95% CI 0.797 to 0.875] for all OSA patients, 0.838 [95% CI 0.792 to 0.883] for males with OSA, and 0.826 [95% CI 0.736 to 0.916] for females with OSA. Wei et al. [34] used VAI as a marker of insulin resistance in OSA patients. They found an AUC of 0.710 (cut-off: 2.10) in normal-weight OSA patients and an AUC of 0.636 (cut-off: 2.66) in overweight/obese OSA patients for predicting insulin resistance in OSA patients.

## Discussion

During the recent decade, the investigation of different lipid parameters and composite lipid indices in OSA patients has become an area of interest for research. In this paper, we determined the relationship between three composite lipid indices, including AIP, LAP, VAI, and OSA. In addition to acceptable AUC for diagnosis of OSA, we found significantly higher levels of these indices in patients with OSA. Although this was observed in individual studies as well, our results can emphasize the need for focus on these indices as the pooled effect was also significant.

All composite lipid indices evaluated in this study showed acceptable performance for the diagnosis of OSA (AUC > 0.7). Despite the fact that AUCs were almost similar for all three indices reported, LAP had a slightly higher AUC. Several studies investigated the diagnostic ability of anthropometric indices (e.g., waist-to-hip ratio (WHR), WC, and BMI) before [37–39]. Wang et al. [37] compared WC, hip circumference (HC), and WHR for the diagnosis of moderate-to-severe OSA and severe OSA. All indices showed inadequate discrimination (AUC < 0.7) for OSA diagnosis. In another study, Lim et al. [38] found WHR superior to BMI and WC in diagnosing OSA (AUC 0.701 for WHR). Kum et al. [39] found that BMI ≥ 31.7 showed an AUC of 0.661 [95% CI 0.625 to 0.696], a specificity of 78.18%, and a sensitivity of 48.13% for diagnosing OSA. Although more studies are required, composite lipid indices showed better discrimination ability compared to traditional anthropometric indices.

OSA, which is defined by repeated upper airway collapse resulting in intermittent hypoxemia, is a significant risk factor for hypertension, heart disease, metabolic syndrome, insulin resistance, impaired glucose tolerance, and the onset of type 2 diabetes mellitus [5, 28]. It is yet unclear how OSA affects lipid metabolism, and the relationship is not straightforwardly linear. Fragmented sleep and chronic intermittent hypoxia (CIH) each individually contribute to hyperlipidemia via a variety of pathways, including adipose tissue inflammation, sympathetic hyperactivity, and oxidative stress [6]. Additionally, sleep disturbances may cause hyperphagia [28]. Only in non-obese patients, OSA besides BMI is related to atherogenic low-density lipoprotein cholesterol (LDL-C) phenotype B [35]. Basoglu et al. found a correlation between greater TG, increased non-HDL-C, reduced HDL-C, and numerous markers of hypoxia, including oxygen desaturation index (ODI), mean and lowest oxygen saturations and time spent with oxygen saturation under 90% (T90) [6].

AIP is believed to show better prediction of cardiovascular outcomes than other single lipid components [7, 28]. Measures of insulin sensitivity are strongly and adversely linked with AIP. As a result, it is a significant index that can indicate improper lipid and glucose metabolism [31]. There is currently proof that higher TG may play a significant part in atherosclerosis. However, HDL-C is diverse and has anti-atherogenic and non-vascular effects. AIP thus represents the equilibrium between atherogenic and protecting lipoproteins. Furthermore, dyslipidemia is a prevalent risk for atherogenesis, which is characterized by low HDL-C or high TG [8].

The following processes are hypothesized to be able to explain the connection between OSA severity and the AIP index. First, CIH could raise TG levels by encouraging the liver's production of TG-related proteins and enzymes. Simultaneously, the elevated sympathetic tone in OSA patients may alter the body's level of endocrine hormones, which may impact lipoprotein lipase synthesis and result in a decrease in serum HDL-C concentration, raising AIP. Furthermore, prior research suggested that blocking the alpha-1 receptor could increase serum HDL-C and lower TG levels. Therefore, hyper-sympathetic tone observed in OSA patients may also contribute to elevated AIP levels [29].

In two sizable male populations from East Asia, Shumizu et al. and Wu et al. demonstrated a link between the TG/HDL-C ratio and OSA. The obvious flaw in these two studies is that they only looked at men, even though gender also plays a role in the relationship between OSA and dyslipidemia, particularly AIP [40, 41]. This ratio was associated with the probability of having severe OSA, and it was more prominent in female patients with severe OSA, according to a study by Fang et al. both men and women could use the TG/HDL-C ratio to forecast their risk of developing severe OSA, but women were more likely to use it. It is likely that severe OSA in women who no longer receive estrogen protection results in more serious dyslipidemia [42].

Another important issue to consider is that the most popular lipid-lowering drugs, statins, may increase HDL-C levels in addition to decreasing total cholesterol; however, this effect varies amongst medications. To resolve this bias, statin-using patients were not included in Bikov et al.'s investigations, and they discovered that this factor did not impact the findings regarding the relationship between AIP and OSA [28].

In 2005, the LAP was first presented which was found to be a more accurate predictor of CVD than BMI. It was then verified as a helpful clinical sign to forecast upcoming cardiovascular events and cardiovascular death. It represents the buildup of lipids in the abdominal region and has been shown to be more accurate at predicting diabetes than BMI [7, 27].

The best methods available for quantifying visceral fat are computed tomography (CT) scan and magnetic resonance imaging (MRI). Given their expensive price, it is challenging to use them broadly. LAP is an easy-to-use indicator that just needs the serum TG level and WC that can distinguish between subcutaneous and visceral adiposity accurately by describing the excessive buildup of fat [5]. There could be several different processes linking LAP to the risk of OSA. First, when compared to other surrogates like the TG/HDL-C ratio, VAI, TG, and the triglyceride-glucose index (TyG), LAP had a better association with the disposal of glucose stimulated by insulin and a higher capacity to identify insulin resistance when compared to insulin sensitivity measured by the hyperinsulinemia euglycemic clamp. Second, beyond LAP evaluated at baseline, alterations in LAP over time, which indicate the effectiveness of lipid-lowering medication and lifestyle modification, have a separate impact on type 2 diabetes mellitus incidence [5].

Based on the Berlin questionnaire, Bianchi et al. showed higher LAP in patients who were at high risk of OSA [27], but Bikov et al. found that a diagnostic sleep test did not support this diagnosis [7]. According to Dong et al.'s findings, LAP was independently correlated with the risk of OSA in patients with type 2 diabetes mellitus [5]. Wei et al. showed that women have considerably higher LAP cutoff levels than males. This might have been caused by the impact of changing hormone levels and amenorrhea in females as they get older. In men with OSA, BMI, and WC were better predictors of insulin resistance [34]. According to Zou et al., LAP had the highest connection with OSA severity, followed by TyG and VAI. LAP's diagnostic efficacy was superior to the Epworth sleepiness scale (ESS) as a tool for OSA diagnosis. However, when employed alone, visceral adiposity indicators did not have a higher diagnostic effectiveness than anthropometric measurements [36].

Recent research demonstrates that imaging methods can accurately measure the visceral fat distribution pattern. Despite CT's ability to be the gold standard for fat distribution discrimination, its high cost and radiation exposure prevent its widespread use in public health and clinical settings. Also, numerous studies have shown that BMI and WC are unreliable for predicting the function and distribution of visceral fat. Determining straightforward metrics that can be consistently used in routine clinical practice as substitute markers of visceral adiposity and indications of elevated cardiometabolic risk have therefore received special attention [30, 43].

VAI, first introduced in 2010, was calculated using several metabolic markers. It was shown to have a strong correlation with metabolic syndrome, insulin resistance, and CVD [7, 30]. In contrast to conventional metabolic indicators, VAI, which included physical and metabolic factors, was more effective than MRI and CT at detecting anomalies in metabolism and abdominal obesity [15, 44].

It has been documented how OSA affects inflammatory indicators. Meanwhile, interleukin-6, high-sensitivity C-reactive protein (hs-CRP), tumor necrosis factor-alpha (TNF-α), and adiponectin levels have been found to be elevated, and visceral adipose tissue (VAT) accumulation and so VAI has been linked to these changes in obese patients. The positive association between TNF-α and VAI was also supported by the evidence that was available [45, 46].

VAI was higher in OSA patients in comparison to controls; however, the increase was only statistically significant in moderate-to-severe OSA, according to Bikov et al. (2022). Additionally, the link between VAI and OSA was no longer significant after controlling for age, gender, and BMI. They believe that this marker may be of limited relevance in clinical practice due to the difficulties of calculating VAI compared to LAP [7]. In Chen et al. investigation, VAI and metabolic score also showed the highest connection with indicators of ODI [30]. In a different study, VAI demonstrated significant correlations with insulin resistance and improved diagnostic accuracy in women with OSA [34]. According to Zheng et al., VAT area and VAI were the two independent risk factors for OSA in individuals with type-2 diabetes mellitus and should be taken into account in the clinical OSA management [47].

In contrast to the aforementioned studies, Mazzuka et al. revealed that there was little association between VAI and the severity of OSA. As blood pressure factor is not included in calculating VAI, unlike metabolic syndrome, and the lack of statistically significant differences in HDL-C with increasing the severity of OSA are all possible explanations for the poor correlation between AHI and VAI and the low sensitivity in OSA patients [20]. According to Zou et al., practically all visceral adiposity and anthropometric indicators were more strongly connected with AHI in women than in men, and the diagnostic accuracy of these indicators was also higher in females. This suggests that OSA and obesity are more closely correlated in females than in males [36].

## Strengths and limitations

The current study is the first that systematically reviewed and analyzed the composite lipid indices in OSA patients. The precise and comprehensive search of major databases and the independent screening of the results by two reviewers made this study strong and reliable. Most of the included studies were cross-sectional or cohort, which allowed for uniform evaluation of study qualification using NOS scaling. There are also some limitations to this study that should be mentioned. First of all, the small number of studies included in the meta-analysis and the small number of subgroups, introduce a potential source of bias. Because of the study design of included papers, no causal relationship can be drawn and the clinical importance of these lipid indices in OSA is better to be evaluated in interventional trials. Second, as a diagnostic tool, some included studies have used polygraphy and some others have used polysomnography which has increased heterogeneity in the results. Another important defect is the evaluation failure of factors like lipid-lowering medications, diet, regular exercise, gender effect, and menopausal status that can affect the OSA severity, in most of the studies included. Fourth, due to the low number of studies reporting sensitivity and specificity, we were not able to conduct meta-analyses for these measures, hence, the results of each individual study were reported descriptively. Finally, composite lipid indices were measured only at the baseline of studies and more frequent evaluation of them could offer more valuable data.

## Conclusion

As OSA is associated with plenty of other comorbidities and could affect remarkably on patients’ quality of life and function, its diagnosis and control are important medical and health issues. According to our results, AIP, LAP, and VAI all are associated with OSA, and also their amounts are progressively higher in higher severities of the disease, so they can be considered valuable diagnostic and prognostic factors for this prevalent disease. It can help clinicians in diagnosing and determining the prognosis of OSA. However, because of the novelty of this topic and the small number of included studies, more research should be conducted to strengthen and support this relevance.

## Supplementary Information

**Additional file 1: Supplementary Table 1.** Search details. **Supplementary Table 2.** Qualities of included studies based on the NOS system. **Supplementary Figure 1.** Sensitivity analysis for meta-analysis of AUCs for diagnosing OSA. **Supplementary Figure 2.** Funnel plot for meta-analysis of AIP between OSA patients and healthy controls. **Supplementary Figure 3.** Sensitivity analysis for meta-analysis of AIP between OSA patients and healthy controls. **Supplementary Figure 4.** Funnel plot for meta-analysis of LAP between OSA patients and healthy controls. **Supplementary Figure 5.** Sensitivity analysis for meta-analysis of LAP between OSA patients and healthy controls.

## Data Availability

Not applicable.
