# Supplementary material for: Composite lipid indices in patients with obstructive sleep apnea: a systematic review and meta-analysis
Source: Lipids Health Dis. 2023 Jun 29;22:84. doi: 10.1186/s12944-023-01859-3 (PMC10308736; doi:10.1186/s12944-023-01859-3)
Supplement: Supplementary file 1 — Additional file 1: Supplementary Table 1. Search details. Supplementary Table 2. Qualities of included studies based on the NOS system. Supplementary Figure 1. Sensitivity analysis for meta-analysis of AUCs for diagnosing OSA. Supplementary Figure 2. Funnel plot for meta-analysis of AIP between OSA patients and healthy controls. Supplementary Figure 3. Sensitivity analysis for meta-analysis of AIP between OSA patients and healthy controls. Supplementary Figure 4. Funnel plot for meta-analysis of LAP between OSA patients and healthy controls. Supplementary Figure 5. Sensitivity analysis for meta-analysis of LAP between OSA patients and healthy controls. [file 12944_2023_1859_MOESM1_ESM.docx]

**Supplementary Materials**

***Supplementary Table 1.*** *Search details*

| **Query** | | **Results**  **(11 May 2023)** |
| --- | --- | --- |
| **PubMed** | | |
| #1 | ("sleep-disordered breathing"[tiab] OR "sleep disordered breathing"[tiab] OR "OSA"[tiab] OR "SAS"[tiab] OR "SDB"[tiab] OR "apnea*"[tiab] OR "apnoea*"[tiab] OR "Apnea"[Mesh] OR "Sleep Apnea, Obstructive"[Mesh]) | 89,309 |
| #2 | (“lipid ind*” OR “atherogenic ind*” OR “VAI”[tiab] OR “visceral adiposity index” OR “LAP”[tiab] OR “Lipid accumulation product” OR "Lipid Accumulation Product"[Mesh] OR “atherogenic coefficient” OR “Atherogenic index of plasma” OR “AIP”[tiab]) | 16,863 |
| #4 | #1 AND #2 | 71 |
| **Scopus** | | |
| #1 | (TITLE-ABS-KEY("sleep-disordered breathing") OR TITLE-ABS-KEY("sleep disordered breathing") OR TITLE-ABS-KEY("OSA") OR TITLE-ABS-KEY("SAS") OR TITLE-ABS-KEY("SDB") OR TITLE-ABS-KEY("apnea*") OR TITLE-ABS-KEY("apnoea*")) | 281,182 |
| #2 | (TITLE-ABS-KEY(“lipid ind*” OR “atherogenic ind*” OR “VAI” OR “visceral adiposity index” OR “LAP” OR “Lipid accumulation product” OR “atherogenic coefficient” OR “Atherogenic index of plasma” OR “AIP”)) | 82,906 |
| #4 | #1 AND #2 | 206 |
| **Web of Science** | | |
| #1 | (TS=("sleep-disordered breathing") OR TS=("sleep disordered breathing") OR TS=("OSA") OR TS=("SAS") OR TS=("SDB") OR TS=("apnea*") OR TS=("apnoea*")) | 267,044 |
| #2 | (TS=(“lipid ind*” OR “atherogenic ind*” OR “VAI” OR “visceral adiposity index” OR “LAP” OR “Lipid accumulation product” OR “atherogenic coefficient” OR “Atherogenic index of plasma” OR “AIP”)) | 121,219 |
| #3 | #1 AND #2 | 229 |
| **Embase** | | |
| #1 | ('apnea, sleep'/exp OR 'apnea, sleep' OR 'apnoea, sleep'/exp OR 'apnoea, sleep' OR 'nocturnal apnea'/exp OR 'nocturnal apnea' OR 'nocturnal apnoea'/exp OR 'nocturnal apnoea' OR 'obstructive sleep apnea'/exp OR 'obstructive sleep apnea' OR 'obstructive sleep apnea hypopnea syndrome'/exp OR 'obstructive sleep apnea hypopnea syndrome' OR 'obstructive sleep apnea syndrome'/exp OR 'obstructive sleep apnea syndrome' OR 'obstructive sleep apnoea'/exp OR 'obstructive sleep apnoea' OR 'obstructive sleep apnoea hypopnoea syndrome'/exp OR 'obstructive sleep apnoea hypopnoea syndrome' OR 'obstructive sleep apnoea syndrome'/exp OR 'obstructive sleep apnoea syndrome' OR 'obstructive sleep-disordered breathing'/exp OR 'obstructive sleep-disordered breathing' OR 'sleep apnea'/exp OR 'sleep apnea' OR 'sleep apnea syndrome'/exp OR 'sleep apnea syndrome' OR 'sleep apnea syndromes'/exp OR 'sleep apnea syndromes' OR 'sleep apnea, obstructive'/exp OR 'sleep apnea, obstructive' OR 'sleep apnoea'/exp OR 'sleep apnoea' OR 'sleep apnoea syndrome'/exp OR 'sleep apnoea syndrome' OR 'sleep apnoea syndromes'/exp OR 'sleep apnoea syndromes' OR 'sleep apnoea, obstructive'/exp OR 'sleep apnoea, obstructive' OR 'sleep disordered breathing'/exp OR 'sleep disordered breathing') | 106,474 |
| #2 | (“lipid ind*” OR “atherogenic ind*” OR “VAI” OR “visceral adiposity index” OR “LAP” OR “Lipid accumulation product” OR “atherogenic coefficient” OR “Atherogenic index of plasma” OR “AIP”) | 40,216 |
| #3 | #1 AND #2 | 171 |
| Total Records | | 677 |
| Total Records after Removing Duplicates | | 505 |

***Supplementary Table 2.*** *Qualities of included studies based on the NOS system*

| Study | Selection | | | | Comparability | Outcome | | Overall  Score |
| --- | --- | --- | --- | --- | --- | --- | --- | --- |
|  | Representation | Sample size | Non-Respondents | Exposure |  | Outcome | Statistical test |  |
| Bianchi et al. (2014) | * | * | * | ** | - | ** | * | 8 |
| Bikov et al. (2021) | * | * | * | ** | - | ** | * | 8 |
| Bikov et al. (2022) | * | * | * | ** | - | ** | * | 8 |
| Cai et al. (2022) | * | * | * | ** | - | ** | * | 8 |
| Cao et al. (2020) | * | * | * | ** | - | ** | * | 8 |
| Chen et al. (2020) | * | * | * | ** | - | ** | * | 8 |
| Dong et al. (2020) | * | * | * | ** | - | ** | * | 8 |
| Kim et al. (2020) | * | * | * | ** | - | ** | * | 8 |
| Mazzuca et al. (2014) | * | * | * | ** | - | ** | * | 8 |
| Meszaros et al. (2021) | * | * | * | ** | - | ** | * | 8 |
| Otelea et al. (2021) | * | * | * | ** | - | ** | * | 8 |
| Wei et al. (2021) | * | * | * | ** | - | ** | * | 8 |
| Wysocki et al. (2016) | * | * | * | ** | - | ** | * | 8 |
| Zou et al. (2020) | * | * | * | ** | - | ** | * | 8 |


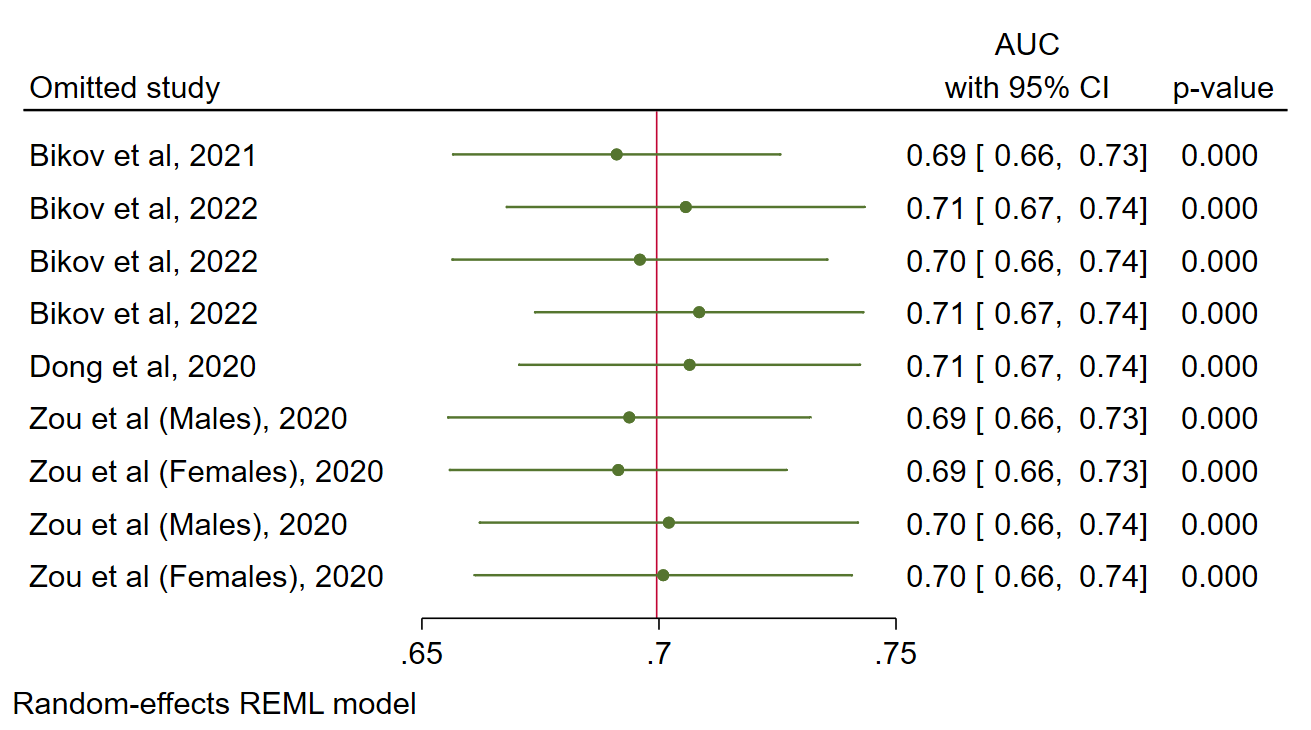


***Supplementary Figure 1.*** *Sensitivity analysis for meta-analysis of AUCs for diagnosing OSA*

*
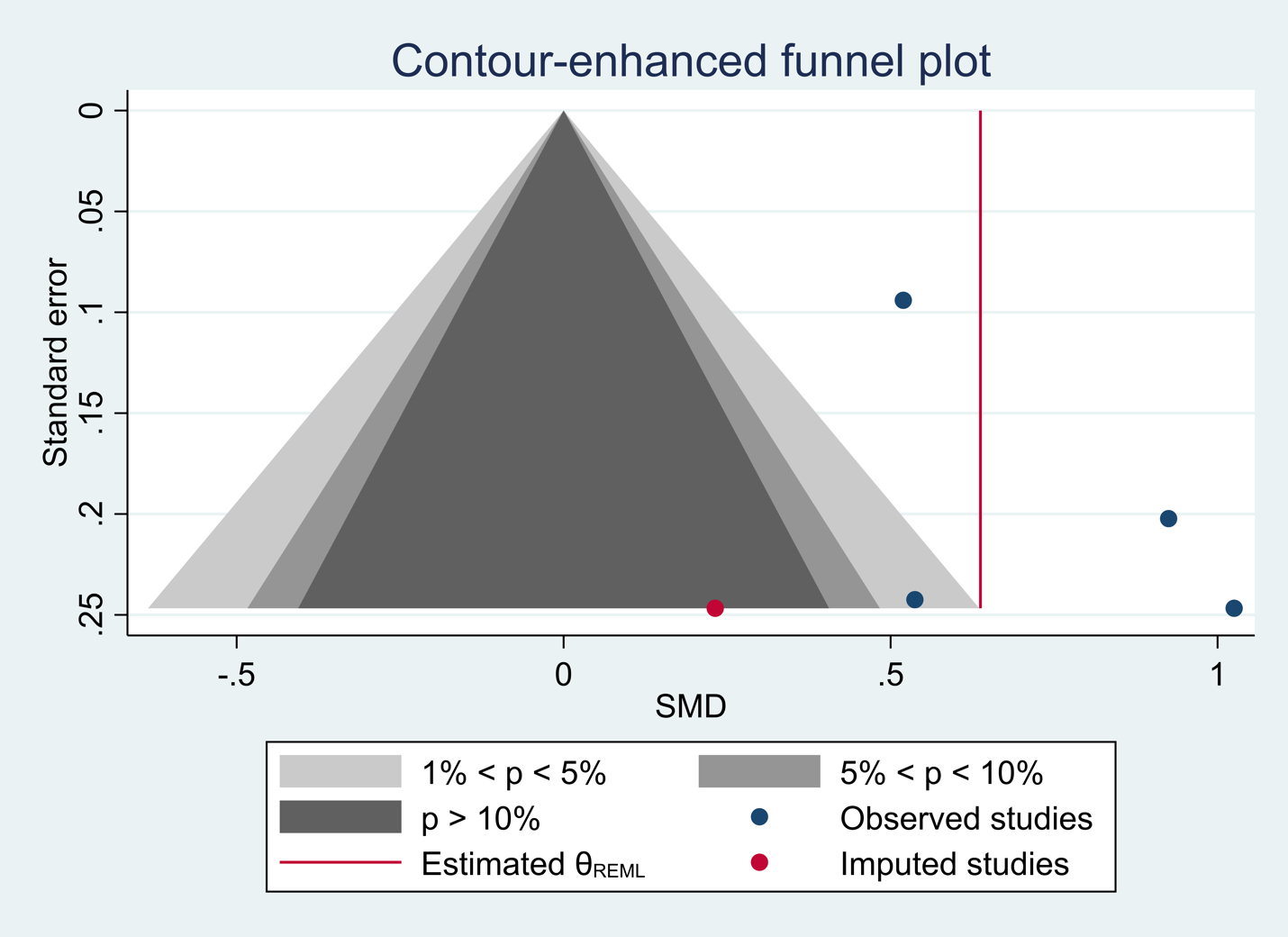
*

***Supplementary Figure 2.*** *Funnel plot for meta-analysis of AIP between OSA patients and healthy controls*

*
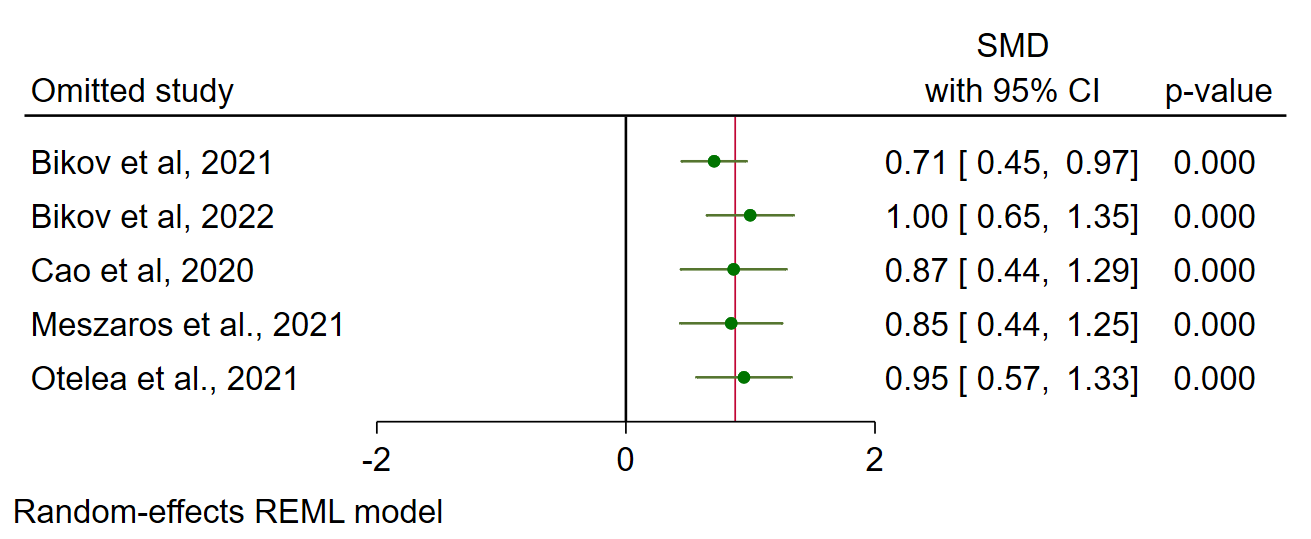
*

***Supplementary Figure 3.*** *Sensitivity analysis for meta-analysis of AIP between OSA patients and healthy controls*

*
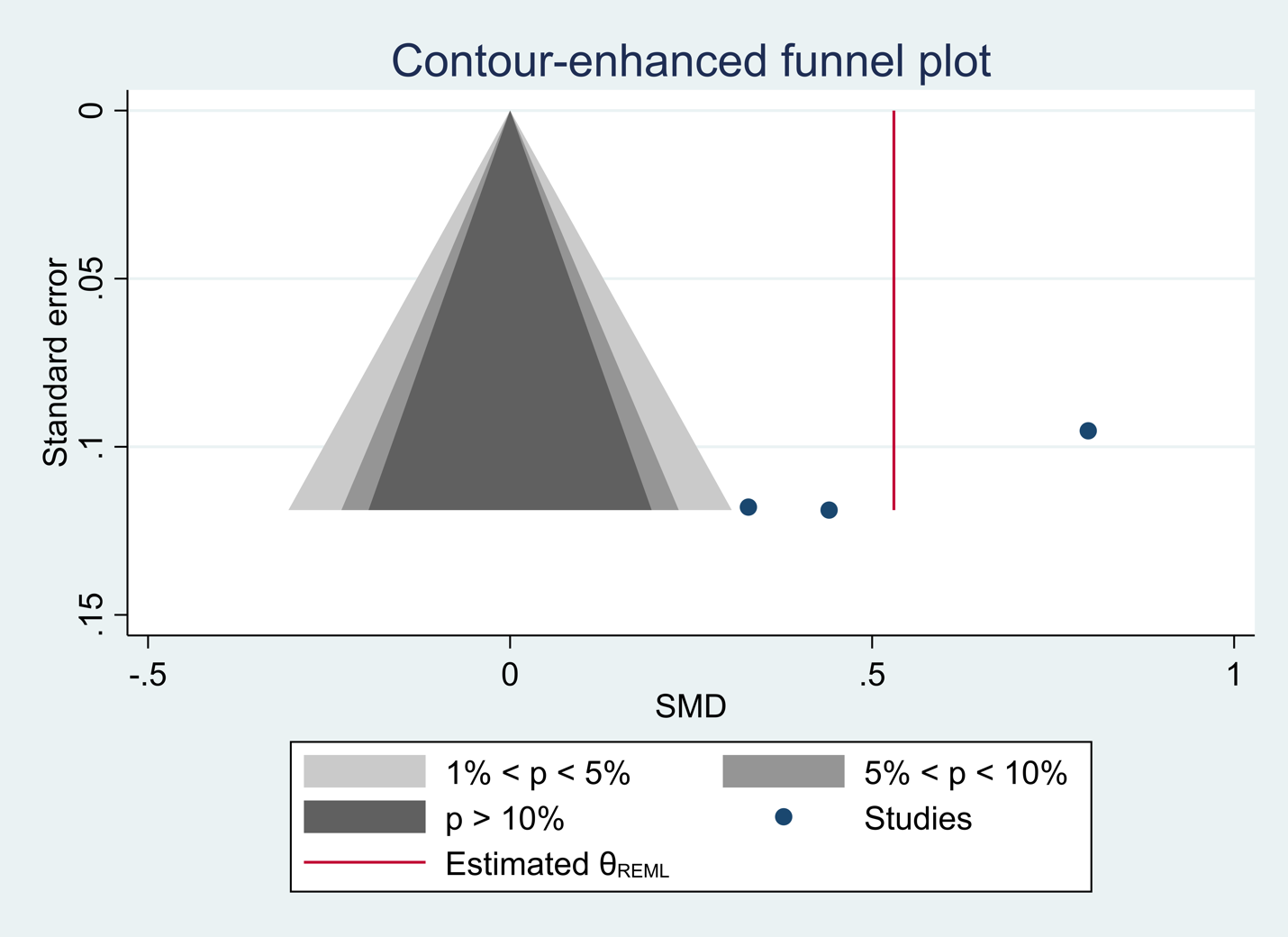
*

***Supplementary Figure 4.*** *Funnel plot for meta-analysis of LAP between OSA patients and healthy controls*

*
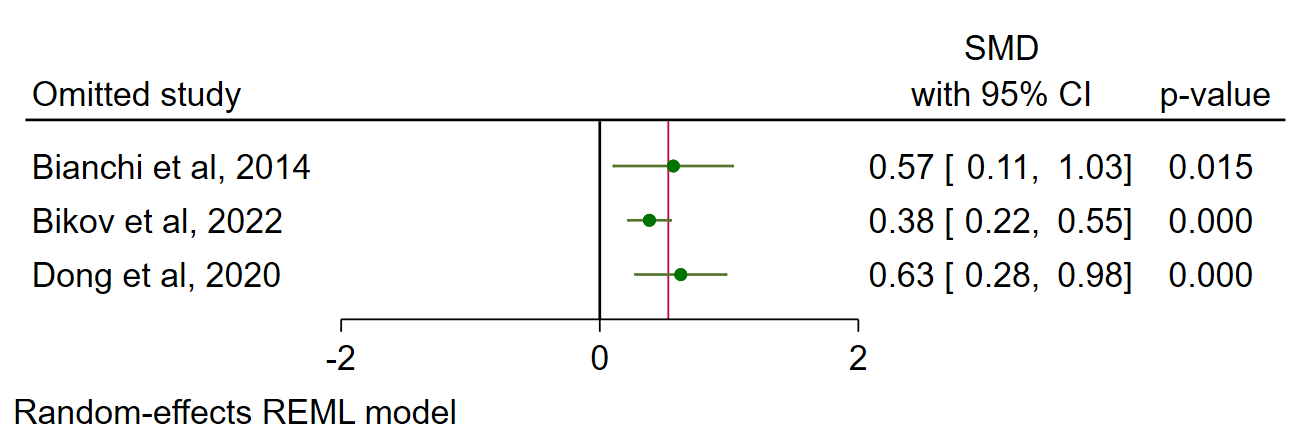
*

***Supplementary Figure 5.*** *Sensitivity analysis for meta-analysis of LAP between OSA patients and healthy controls*
